# HDL, heart disease, and the lung

**DOI:** 10.1016/j.jlr.2022.100217

**Published:** 2022-04-27

**Authors:** Kathrin Frey, Arnold von Eckardstein

**Affiliations:** University Hospital Zurich and University of Zurich, Institute of Clinical Chemistry, Zurich, Switzerland

**Keywords:** ASCVD, atherosclerotic cardiovascular diseases, DPPC, dipalmitoylphosphatidylcholine

For more than 50 years, a low plasma level of HDL-cholesterol has been known as an independent risk factor of atherosclerotic cardiovascular diseases (ASCVD). In addition, HDL particles exert a plethora of potentially anti-atherogenic activities on many cells including endothelial cells, smooth muscle cells, as well as monocyte-derived macrophages and other inflammatory cells. Nevertheless, therapeutic interventions raising HDL-cholesterol did not improve the prevention of cardiovascular events beyond standard therapy with statins. Moreover, Mendelian randomization studies ruled out genetic causality of the inverse association between HDL-cholesterol and risk of ASCVD (1). These findings led to two opposing interpretations: according to the first, the association of low HDL-cholesterol with ASCVD does not reflect any direct anti-atherogenic role of HDL but indirectly the pro-atherogenic impact of the frequently confounding hypertriglyceridemia, insulin resistance, or inflammation. This view was substantiated by clinical guidelines, which abandoned HDL-cholesterol as a therapeutic target. The antithesis is built on the fact that HDLs do not exert their biological activities by their cholesterol content (i.e., HDL-cholesterol) but as whole particles or specific proteins or lipids, which are carried by HDL and not mirrored by HDL-cholesterol. As the consequence, functional HDL biomarkers are searched and validated toward their prognostic performance in clinical and epidemiological studies. Candidates include numbers of total and distinct HDL particles, distinct proteins, lipids, and even noncoding RNAs (1).

More than 40 previously published mass-spectrometric studies found about 300 different proteins associated with HDL (2). Several of them were either enriched or depleted in HDL of patients with ASCVD, diabetes, or other diseases in comparison to HDL of healthy control subjects (2). Among them, APOC3 is probably the best investigated example. In four prospective studies, APOC3-free HDL but not APOC3-containing HDL showed the classical inverse association with incident cardiovascular events (3). Moreover, the presence of APOC3 was found to adversely affect the ability of HDL to inhibit the apoptosis of endothelial cells and to promote cholesterol efflux from macrophages (4, 5). Genetic population studies indicate genetic causality between APOC3 plasma levels and risk of ASCVD (6, 7). However, these studies did not dissect whether this association is driven by the adverse role of APOC3 in the metabolism of triglyceride-rich lipoproteins or in the functionality of HDL or both.

A less prominent example is pulmonary surfactant protein B (gene name: *SFTPB*; protein name: PSPB; short name: SP-B), which was previously found enriched in HDL from patients with ASCVD or diabetes mellitus type 2 (4, 8) and to predict mortality in patients with type 2 diabetes on hemodialysis (9) or heart failure (10). In the current issue of *Journal of Lipid Research*, Shao *et al.* (11) provide further evidence for the potential of SP-B as an HDL-associated biomarker, namely for risk of ASCVD in patients with diabetes mellitus type 1. In a cohort of 145 type 1 diabetes patients randomly selected from all subjects within the Coronary Artery Calcification in Type 1 Diabetes (CACTI) study, 47 subjects developed an ASCVD event within an average follow-up of 16 years. Isotope dilution parallel reaction monitoring analysis of HDL isolated by ultracentrifugation revealed five proteins to differ by abundance between subjects with and without incident cardiovascular disease events. Three of them, namely SP-B, α-1-microglobulin/bikunin precursor, and insulin-like growth factor I, were significantly associated with ASCVD incidence upon Cox proportional hazard analysis. After correction for classical ASCVD risk factors and potential confounders, SP-B was the only one, which remained strongly associated with incident ASCVD events. Of note, the association was independent of smoking, which was previously shown to cause increases in SP-B plasma levels (12). Also of note, the association was independent of HDL-cholesterol although the authors detected SP-B almost exclusively in HDL (11).

SP-B is a positively charged hydrophobic protein with a molecular weight of approximately 8 kDa. It is derived from a proteolytically cleaved large precursor, expressed by lung type II epithelial cells. Together with three other surfactant-associated proteins (SP-A, SP-C, SP-D), SP-B assembles with dipalmitoylphosphatidylcholine (DPPC) and other phospholipids in lamellar bodies that are stored intracellularly to be eventually secreted into the alveolar airspace (13, 14). There the lamellar bodies form a network of organized membranes termed tubular myelin between the hypophase, a thin aqueous layer on the alveolar epithelial cell surface, and the air. As a surface-active agent, the pulmonary surfactant prevents alveolar collapse at low lung volume after expiration but also contributes to the prevention of lung infections and injuries (13, 14). Occasionally, some lamellar bodies are taken up into both type II epithelial cells and alveolar macrophages. In the former, they become part of the intracellular storage pool for later resecretion (13, 14). From the latter, cholesterol is effluxed with the help of ATP-binding cassette transporters ABCA1 and ABCG1 and the apoA-I binding protein AIBP (15).

Structural and functional aberrations of surfactant were reported in the context of a wide range of pulmonary morbidities, including obstructive, interstitial, and infectious lung diseases (13, 14) but also in heart failure (14). Plasma levels of SP-B correlate with the severity of dyspnea and the prognosis in patients with chronic heart failure (14). Thus, the association of SP-B with ASCVD events may be a noncausal reporter of cardiopulmonary dysfunctions, which ultimately cause the increased risks for ASCVD events. In this line, Shao *et al.* suggest that the enrichment of HDL with SP-B indicates capillary leakage, which is increased in diabetes mellitus, not only in the lung but in many organs including the heart and kidney. It would hence have been interesting to see how measures of pulmonary and cardiac function such a VO_2_max and B-type natriuretic peptides affect the association of SP-B with ASCVD events in the study of Shao *et al.* (11).

As an alternative explanation, the enrichment of HDL with SP-B per se or together with other surfactant components may cause the loss of physiological functions or gain of pathological functions which ultimately contribute to ASCVD. However, Shao *et al.* (11) did not find any significant impact of SP-B on the capacity of HDL to promote cholesterol efflux from macrophages or to inhibit the expression of adhesion molecules in endothelial cells. Neither did our lab find any association of SP-B abundance with cholesterol efflux capacity nor anti-apoptotic effects of HDL toward endothelial cells (16). However, Banfi *et al.* (17) found that the enrichment of HDL with immature SP-B impairs the antioxidant capacity of HDL. Moreover, one cannot rule out that other not yet investigated HDL functions are impaired, by either SP-B itself or lipid components of the surfactant that are transferred together with SP-B to HDL. In view of the inverse association of HDL-cholesterol with several lung diseases as well as the protective effects of *APOA1* overexpression or HDL mimetics in animal models of acute respiratory distress syndrome or pneumonia (18, 19), it will be very interesting to investigate the effects of HDL/SP-B interactions in bioassays that model pathogenic mechanisms in lung diseases.

After fractionation of plasma into the lipoprotein classes and lipoprotein-free plasma, Shao *et al.* recovered SP-B almost exclusively in the HDL fraction. This raises two questions on its structural and metabolic basis.

First, is SP-B physically associated with HDL or part of distinct surfactant particles that share the density of HDL and are therefore only coisolated by ultracentrifugation? With densities of 1.06 kg/l and less than 1.05 kg/l lamellar bodies and tubular myelin, respectively, are lighter than HDL (20). Moreover, Kopecky *et al.* (9) measured SP-B with a sandwich ELISA, which utilized an anti-apoA-I antibody to capture HDL and an anti-SP-B antibody for quantification. Thus, it is very likely, that in the plasma compartment SP-B is a component of HDL rather than of a coisolated non-HDL particle.

Second, where does SP-B associate with HDL and how does it then reach the circulation? In principle, SP-B or SP-B-containing lamellar bodies can associate with HDL particles either in the circulation after leakage through the capillaries or within the alveolar airspace. The former scenario is assumed by Shao *et al.* (11). However, the latter scenario cannot be ruled out, since apoA-I is recovered in bronchoalveolar lavage fluids (21). At least in mice, *Apoa1* was even found expressed by several cells of the lung including type II epithelial cells. In humans, however, this was only found in a limited period during late embryonic development (22). It will be hence interesting to investigate the proteome and lipidome of apoA-I-containing particles of bronchoalveolar lavage fluids to unravel if these alveolar HDL particles contain components of surfactant such as SB-P and DPPC and also other lung-protective proteins such as alpha1-antitrypsin (23).

The finding of SP-B but also DPPC, that is the characteristic lipid component of surfactant, in HDL indicates that the lung contributes to the remodeling and metabolism of HDL. This is also indicated by the expression of several genes in the lung that are relevant in HDL metabolism, notably *APOA1*, *ABCA1*, and *ABCG1*. In mice, the knock-out of these genes generates pulmonary phenotypes (18). Vice versa, overexpression of *APOA1* or treatment with reconstituted HDL showed positive effects in animal models of several lung diseases such as acute lung injury, acute respiratory distress syndrome, pneumonia, asthma, chronic obstructive lung disease, pulmonary fibrosis, or pulmonal hypertension (18). This suggests that HDL is relevant for pulmonary integrity and function.

In conclusion, Shao *et al.* corroborated previous findings that the enrichment of HDL with SP-B predicts adverse cardiovascular outcomes. They also showed that in plasma, SP-B is almost entirely transported by HDL. These two observations together point to the lung as both a player and a target of HDL’s metabolism and function.
